# Comparison of near infrared spectroscopy (NIRS) and near-infrared transillumination-backscattering sounding (NIR-T/BSS) methods

**DOI:** 10.1038/s41598-020-75037-1

**Published:** 2020-10-29

**Authors:** Agnieszka Gruszecka, Marcin Gruszecki, J. Patrick Neary, Jyotpal Singh, Taylor Teckchandani, Monika Waskow, Magdalena Wszedybyl-Winklewska, Wojciech Guminski, Andrzej F. Frydrychowski, Jacek Rumiński, Piotr Lass, Gregory P. Kratzig, Pawel J. Winklewski

**Affiliations:** 1 Department of Radiology Informatics and Statistics, Medical University of Gdansk, Tuwima Str. 15, 80-210, Gdansk, Poland; 2 Faculty of Kinesiology and Health Studies, University of Regina, Regina, Canada; 3 Institute of Health Sciences, Pomeranian University of Slupsk, Slupsk, Poland; 4 Department of Human Physiology, Medical University of Gdansk, Gdansk, Poland; 5 Department of Computer Communications, Faculty of Electronics, Telecommunications and Informatics, Gdansk University of Technology, Gdansk, Poland; 6 NIRTI SA, Wroclaw, Poland; 7 Department of Biomedical Engineering, Faculty of Electronics, Telecommunications and Informatics, Gdansk University of Technology, Gdansk, Poland; 8 Department of Nuclear Medicine, Medical University of Gdansk, Gdansk, Poland; 9 Royal Canadian Mounted Police Depot Division, Regina, SK Canada

**Keywords:** Physiology, Medical research

## Abstract

The aim of the study was to compare simultaneously recorded a NIR-T/BSS and NIRS signals from healthy volunteers. NIR-T/BSS is a device which give an ability to non-invasively detect and monitor changes in the subarachnoid space width (SAS). Experiments were performed on a group of 30 healthy volunteers (28 males and 2 females, age 30.8 ± 13.4 years, BMI = 24.5 ± 2.3 kg/m^2^). We analysed recorded signals using analysis methods based on wavelet transform (WT) for the wide frequency range from 0.0095 to 2 Hz. Despite the fact that both devices use a similar radiation source both signals are distinct from each other. We found statistically significant differences for WT amplitude spectra between both signals. Additionally, we showed different relationships of both signals to blood pressure. Collectively, based on the present findings and those of previous studies, we can conclude that the combination of NIR-T/BSS or NIRS signals and time–frequency analysis opens new frontiers in science, and give possibility to understand and diagnosis of various neurodegenerative and ageing related diseases to improve diagnostic procedures and patient prognosis.

## Introduction

Cerebral haemodynamic is incredibly complex. The maintenance of cerebral blood flow (CBF) is paramount for proper brain function, and CBF is determined by cerebral perfusion pressure and conductance through the cerebral vasculature^1^. Despite many years of intensive research, models which describe dynamical processes in the brain are relatively unknown. However, CBF can be adjusted by changes in perfusion pressure, the metabolic activity of the brain, intrinsically by humoral factors, and the autonomic nervous system. Thus, an understanding of the factors that contribute to cerebral haemodynamics in normal health will aid our understanding of the mechanisms in a wide range of neurological diseases, such as traumatic brain injury or stroke.

To better understand the integrative cerebral processes that alter flow, pressure and metabolism, new technological advances are needed. Recently, the ability to non-invasively detect and monitor changes in the subarachnoid space width (SAS), which begin to address perfusion pressure and cerebrospinal fluid pulsatility, has been developed^2–4^. Using near-infrared transillumination-backscattering sounding (NIR-T/BSS), it was shown that oscillations of SAS width could be a potential marker of cerebrospinal fluid pulsatility^5^. A single sensor-detector module of NIR-T/BSS consist of the source (S) and two photo-detectors (PD—proximal detector and DD—distal detector). Based on Monte-Carlo simulations^4^, the optimal distances for both detectors from the source were chosen (PD—7 mm and DD—28 mm). The short distance between source and detectors limits extracranial contamination^6,7^. From the source, the near infrared radiation is emitted (wavelength 880 nm). It was shown that the infrared radiation at wavelength 880 nm easily penetrates tissues and is almost completely insensitive to changes in haemoglobin oxygen saturation^8–10^. The radiation penetrates the skin, skull and tissue layers, propagates through the SAS, and returns to the detectors^3^. To eliminate the absorption from the skin and scalp bone, the signal from the PD is used^2^. The transillumination quotient (TQ), which is a ratio of the DS to PS signals, is sensitive to changes in the width of the SAS^2^. More recently, Monte Carlo simulations were performed to illustrate that for the chosen source-detector distances^11^, the dominant contribution of the NIR-T/BSS signal is SAS width changes rather than the absorption of the brain. Frydrychowski et al.^12^ showed high interdependence (r = 0.81, p < 0.001) between NIR-T/BSS signal and SAS width changes measured with magnetic resonance imaging. In turn, Winklewski et al.^13^ used the NIR-T/BSS system to study SAS width oscillations at cardiac and respiratory frequencies. Further validation studies of the NIR-T/BSS have demonstrated the dynamics of the SAS width within a wide range of frequencies from 0.0095 to 2 Hz^3^.

A widely recognized method which enables detection of cerebral haemodynamic is near-infrared spectroscopy (NIRS). NIRS is a spectroscopic method that uses the near-infrared region of the electromagnetic wave spectrum (from 700 to 3000 nm). In general, NIRS enables continuous, non-invasive measurements of the relative changes in oxygenated haemoglobin, deoxygenated haemoglobin or total haemoglobin. NIRS is based on the modified Beer-Lambert law which relates the attenuation of light to the properties of the material through which the light is passes^14,15^. It was shown that oxygenated and deoxygenated haemoglobin have different absorption characteristics in the near-infrared range. To determine an average, the local oxygen saturation of the haemoglobin measured absorption can be used.

NIR-T/BSS is distinct from the NIRS despite the fact that both devices use a similar radiation source. First, NIRS uses several wavelengths (for the detection of deoxy- and oxy-haemoglobin; 760, 850 nm, respectively), while NIR-T/BSS use only one. Second, frequency modulation of the source in the NIR-T/BSS is much less than in NIRS. Most important is the fact that any physiological disturbances are immediately visible in NIR-T/BSS, while in NIRS they appear with some delay.

The aim of the study was to compare simultaneously recorded a NIR-T/BSS and NIRS signals from healthy volunteers. This could provide more knowledge about the properties of these signals and thus could be valuable for the management of neurocritical care of patients, and may contribute to a more effective planning of therapeutic strategies. To avoid any interference between simultaneously registered signals, the NIR-T/BSS’s signal was recorded from left hemisphere, and NIRS from the right. A mathematical method based on wavelet transform was used to find other differences between those two signals. To the best of our knowledge, this is the first study comparing those two signals.

## Results

During the experiment simultaneous measurements of blood pressure (BP), SAS from the left pre-frontal cortex (SAS_LEFT_), and HbO_2_ from the right pre-frontal cortex were recorded. Subject characteristics are shown in Table 1.

**Table 1:** Participant demographics and average physiological responses during the 30 min supine rest condition.

Age (years)	30.8 ± 13.4
BMI (kg/m^2^)	24.5 ± 2.3
HR [beats/min]	60.6 ± 10.8
DBP [mmHg]	68.16 ± 9.65
SBP [mmHg]	119.67 ± 14.51
MAP [mmHg]	85.32 ± 10.88
SaO_2_ [%]	97.81 ± 0.61
PETCO_2_ [mmHg]	32.95 ± 3.16

The correlation between the SAS and HbO_2_ was estimated to search for the relationship between both signals. The estimated correlation (r = 0.11 ± 0.07, p < 0.001) was very low between those two signals.

**Figure 1:**
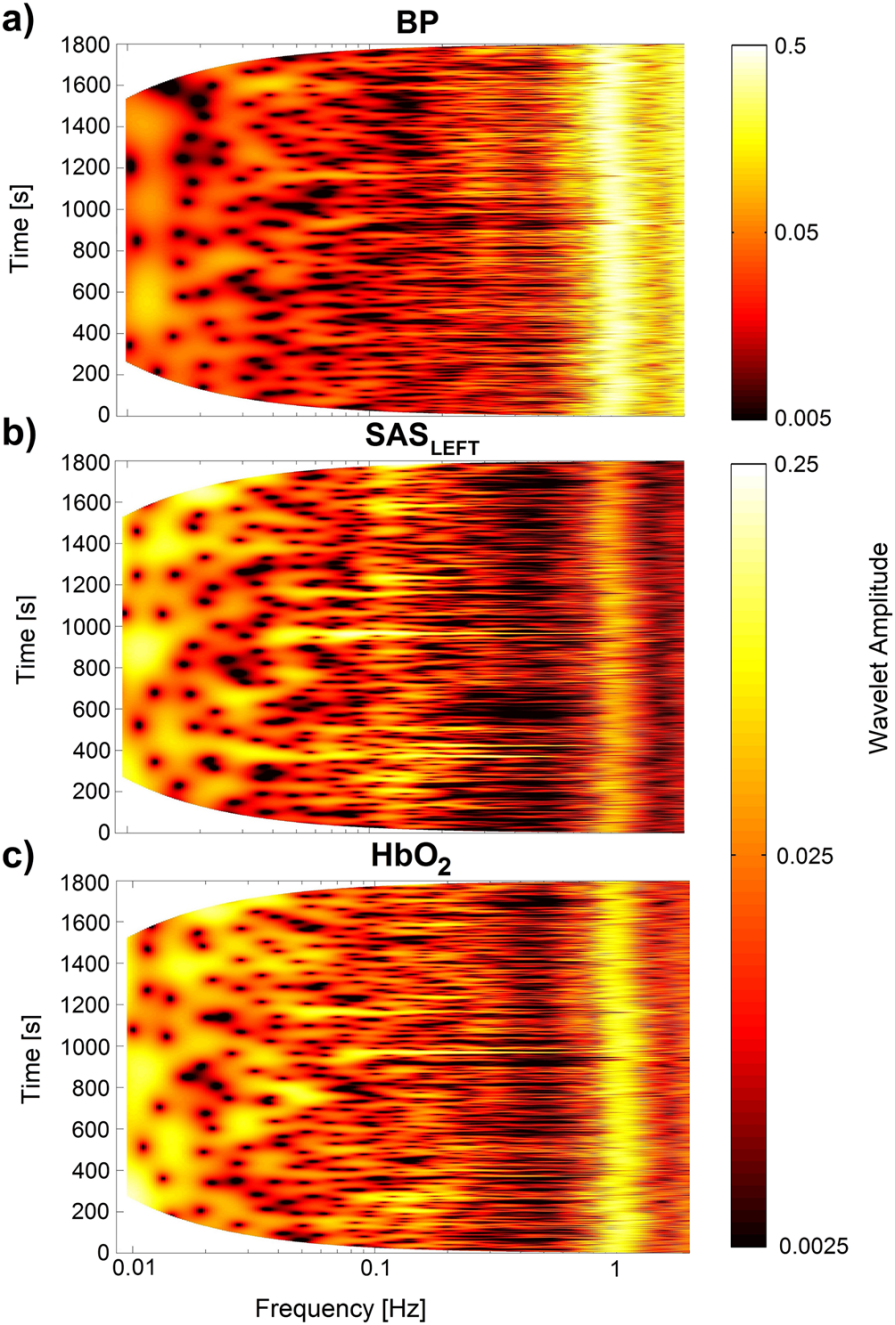
Wavelet transform of recorded signals: BP (**a**), SAS_LEFT_ (**b**) and HbO_2_ (**c**) for one representative volunteer.

Figure 1 shows the result of applying the wavelet transform (WT) for all three recorded signals. It is clearly visible that all three oscillations manifest over the wide frequency range from 0.0095 Hz to 2 Hz in recordings of 30 min duration. For all three signals we observed a similar cardiac component with a frequency about 1 Hz. Additionally, it is evident that SAS and HbO_2_ signals (Fig. 1b,c) have more low frequency components (below 0.03 Hz) than the BP signal (Fig. 1a).

**Figure 2:**
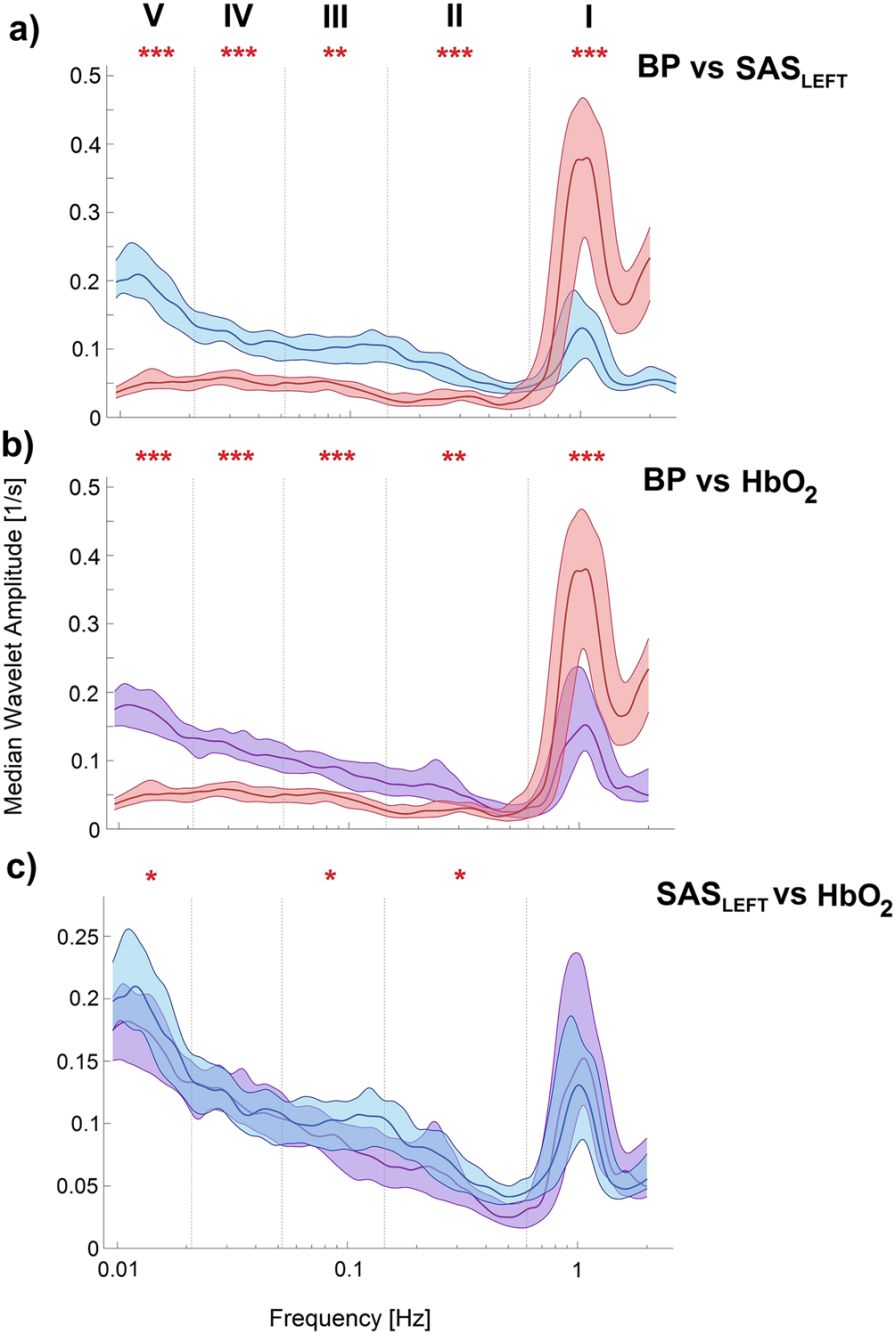
Comparison (BP vs. SAS_LEFT_ (**a**), BP vs. HbO_2_ (**b**) and SAS_LEFT_ vs. HbO_2_ (**c**)) of medians (thick lines) of the time-averaged wavelet transforms of signals recorded in all 30 subjects: BP (red line), SAS_LEFT_ (blue line) and HbO_2_ (violet line) obtained from 30 min recordings. Shaded areas indicate the inter-quartile range (25th, 75th percentiles). *p < 0.05; **p < 0.01; ***p < 0.001.

To simplify the comparison of estimated WT amplitude in terms of their frequency content, Fig. 2 was plotted. Figure 2 illustrates the comparison of the median time-averaged amplitude of wavelet transforms between all measured signals. Frequency axis was divided into six intervals. Each interval is associated with various physiological functions. Intervals I (0.6–2 Hz) is related to cardiac activity. Second interval (0.145–0.6 Hz) correspond to respiratory function. Third interval (0.052–0.145 Hz) corresponds to smooth muscle cell activity. According to Stefanovska et al.^16^ studies interval IV (0.021–0.052 Hz) is associated with autonomic innervation of smooth muscle. The last intervals V (0.0095–0.021 Hz) are related to endothelial functions nitric oxide (NO) dependent^17^ (Kvandal et al. 2006, Shiogai et al. 2010). Bernjak et al.^18^ showed that the oxygen saturation recordings reflect the non-autonomous character of the oscillatory processes described above. Gruszecki et al.^3^ showed that all of the mentioned oscillations are transmitted to the CSF, affecting its circulation and resulting in SAS width changes. The most significant differences (p < 0.001) were observed between BP vs. SAS_LEFT_ spectra (Fig. 2a), and BP vs. HbO_2_ spectra (Fig. 2b) for almost all frequency intervals measured. For the SAS_LEFT_ vs. HbO_2_ spectra (Fig. 2c) the significant differences (p < 0.05) were observed for respiration, myogenic and endothelial (NO dependent and independent) frequency intervals.

**Figure 3:**
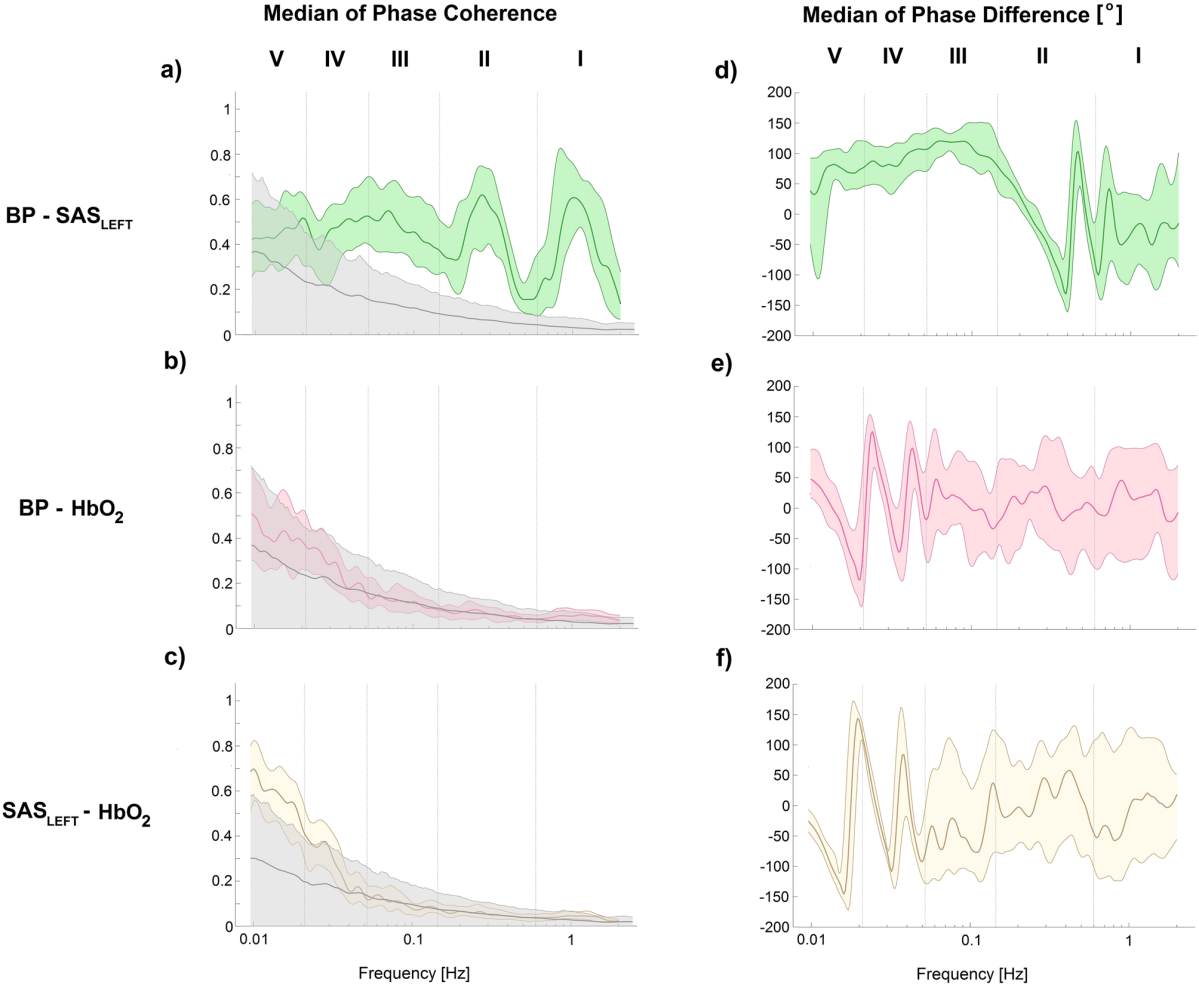
Median (thick lines) of wavelet phase coherence between (**a**) BP vs. SAS_LEFT_, (**b**) BP vs. HbO_2_ and (**c**) SAS_LEFT_ vs. HbO_2_. (**d**–**f**) Phase differences for the coherence in (**a**–**c**). Coloured shading indicates the interquartile range (25th, 75th percentiles) for 30 subjects. Coherence below the 95th percentile of the surrogates (light grey line and shading) is not considered significant.

Figure 3 shows wavelet phase coherence and phase difference between collected signals. If value of phase coherence was higher than 95th percentile of 435 (2-permutation of 30 subjects) inter-subject surrogate then its value was significant. Phase difference at each frequency was considered only for significant phase coherence. From Fig. 3a it is visible that phase coherence is significant for myogenic, respiration, and cardiac frequency intervals. If phase difference for BP and SAS_LEFT_ was positive (negative) then the SAS_LEFT_ (BP) signal is leading. At the respiratory (II) and cardiac (I) frequency intervals, the mean value of phase difference is equal to zero. It means that there is no leading signal. For the myogenic interval, the value of phase difference was positive so the SAS_LEFT_ signal was leading (Fig. 3d). The same results were obtained by Gruszecki et al.^3^. We did not observe any significant coherence for BP and HbO_2_ signals for all frequency intervals (Fig. 3b), thus we cannot consider phase difference between those two signals (Fig. 3e). Statistically significant coherence differences between SAS_LEFT_ and HbO_2_ were obtained for low frequencies in the V frequency interval (Fig. 3c). The negative (positive) value of phase difference for SAS_LEFT_ and HbO_2_ indicates that the phase of the SAS_LEFT_ (HbO_2_) is leading. For the endothelium frequency intervals (V), the mean value of phase difference is equal to zero which indicates that there is not leading signal (Fig. 3f).

## Discussion

This study investigated the relationship between the NIR-T/BSS and NIRS signals. We estimated the correlation between these two signals. It was found that the linear correlation is very low, and thus the signals are hardly related. These results are not surprising because both signals show different physiological parameters. NIRS measures relative changes of haemoglobin oxygenation while NIR-T/BSS measures changes in the width of the subarachnoid space width.

Next, we investigated how NIRS and NIR-T/BSS signals were related to the blood pressure. Using wavelet transform, we found that the WT amplitude of both signals were significantly different from WT amplitude of BP across all considered frequency ranges (Fig. 2a,b). These results are consistent with previous studies^3,19^. Additionally, similar analysis for NIRS and NIR-T/BSS spectra also showed some statistically significant differences in four frequency intervals: respiration, myogenic and endothelial NO dependent and independent (Fig. 2c). This provides evidence that different physiological mechanisms may have a differential impact on SAS and haemoglobin oscillations.

Additionally, we also investigated how NIRS and NIR-T/BSS signals are related to blood pressure using wavelet phase coherence (see Fig. 3a,b). Significant coherence was found between BP signal SAS signals in the cardiac, respiration, and myogenic intervals (Fig. 3a). Phase difference analysis suggests that at the respiration and cardiac frequencies both signals are independent. This indicates that the heart and lungs are responsible for the generated oscillations (Willie et al. 2014). In the myogenic frequency, the leading phase comes from SAS signal (Fig. 3d). This may indicate active local processes adjusting vessel activity to the metabolic requirements of the brain^20^. All of this is consistent with studies of Gruszecki et al.^3^. In contrary, phase coherence between BP and HbO_2_ signals are not statistically significant across considered frequency range (Fig. 3b). This is consistent with studies of Cui et al.^19^ and could indicate that both oscillators exhibit considerable variability and this might result in low, none statistically significant coherence values.

Significant phase coherence between NIRS and NIR-T/BSS signals was observed for low frequencies (V frequency interval—see Fig. 3c). Phase difference analysis suggests that at these frequency intervals both signals are independent and oscillations are generated centrally by the endothelium. This may indicate that the endothelium plays the most significant role in generation of oscillations in cerebral haemodynamics.

Big disproportion of male (28) and female (2) number of subjects might be considered as the main limitation of the study. However, we demonstrated previously that there were no significant differences due to gender in the wavelet transforms or in the phase coherence analysis^3^.

Our study provides additional proof that NIR-T/BSS signal is distinct from the NIRS signal despite the fact that both devices use a similar radiation source. We analysed both signals using analysis methods based on wavelet transform for the wide frequency range from 0.0095 to 2 Hz. We found statistically significant differences for WT amplitude spectra between both signals. Additionally, we showed different relationships of both signals to blood pressure. Collectively, based on the present findings and those of previous studies, we can conclude that the combination of NIR-T/BSS or NIRS signals and time–frequency analysis opens new frontiers in science, and that these methodological techniques may assist in the diagnosis of various neurodegenerative and ageing related diseases. Ultimately, this will assist to improve our diagnostic abilities to enhance patient care and prognosis.

## Materials and methods

### Subjects

The experiment was performed with a group of 30 healthy, non-smoking volunteers (28 males and 2 females, age 30.8 ± 13.4 years, BMI = 24.5 ± 2.3 kg/m^2^). The experiment was carried out in accordance with the recommendations of Helsinki for the ethical conduct of human subjects. The experimental protocol and the study were approved by an institutional Research Ethics Board in Regina (REB#2017–013). All volunteers were informed in detail about the study’s objectives and any potential risk to their health. All subjects gave written informed consent to participate in the study. Participants were asked to refrain from coffee, tea, cocoa, nicotine and any food and beverages containing methylxanthine for at least 12 h, and no alcohol at least 24 h before the experiment.

### Experimental design

All tests were conducted in a comfortable quiet room pre-set to a temperature of 18–20 °C with low ambient light. Before starting the experimental protocol, the participant was asked to void their bladder within 30 min. Thereafter, the participant was instrumented with the research equipment (see below), and then was instructed to lay in a supine position for 30 min. Participants were instructed to “not move their eyebrows excessively, remain quiet, and not fall asleep” for 30 min during supine rest. A blanket was provided as an option to use to keep the participant warm, and a pillow used to reduce any strain on the neck musculature. All auditory and visual distractions were minimized.

### Measurements

Blood pressure (BP) and heart rate (HR) were measured using a Finapres NOVA (Finapres Medical Systems, Arnhem, The Netherlands). Finger BP was calibrated against brachial arterial pressure and the signal was collected from left index finger using photoplethysmography. HR was determined from an electrocardiogram (ECG) signal. The ECG ground electrode was placed on the left anterior superior iliac spine and the two main leads under the middle portion of each clavicle (Lead I). Oxyhaemoglobin saturation (SaO_2_) in the blood was measured using a Nellcor PM10N Portable SpO_2_ Patient Monitoring System (Medtronic Canada, Vancouver, BC) placed on the right index finger. The participant wore a nose-clip and mouthpiece to collect expired gas samples for end-tidal CO_2_ (PETCO_2_) analysis. The gas analyzers were calibrated using primary standard gases (16.0% O_2_, 4.0% CO_2_, balance N_2_) before all assessments. The NIRS signal was collected using a PortaLite system (Artinis Medical, The Netherlands), with data collected at 10 Hz from pre-frontal cortex of the right hemisphere. The PortaLite system contains transmitters at 30, 35 and 40 mm from the receiver, which allows a penetration depth of approximately one-third to on-half of the distance between optodes^21^. During the experiment we registered the following NIRS signals: relative changes in oxy- (HbO_2_), deoxy- (HHb), and total haemoglobin (tHb = HbO_2_ + HHb), and haemoglobin difference (Hb_diff_ = HbO_2_-HHb). The NIRS sensor was carefully secured with a tensor bandage wrapped around the forehead while ensuring no admission of background light. To avoid any interference between NIRS and NIR-T/BSS signals we recorded only SAS_LEFT_ from left hemisphere using the SAS Monitor (NIRTI SA, Wroclaw, Poland).

To be sure that we analyse signals from brain vessels, we analysed NIRS signal that penetrate the deepest regions. We did not observe any statistically significant differences between the amplitude spectra of wavelet transform between all registered NIRS signals for the same depth penetration. Thus, in the current study we decided to analyse cerebral oxyhaemoglobin (HbO_2_) as many others^18,19,22^.

All measured signals were simultaneously recorded for 30 min. The powerlab (AD Instruments, Colorado Springs, Colorado, USA) and LabChart Pro were used to import and view collected signals, respectively. All signals before analysis were downsampled to 10 Hz, detrended using a moving average with a window size of 120 s and normalized by subtraction of their mean and division by their standard deviation.

### Wavelet transform

To analyse collected signals which changes with time caused by physiological perturbations was used the wavelet transform. The wavelet transform is defined as:

$$W\left( {s,t} \right) = \frac{1}{\sqrt s }\mathop \smallint \limits_{ - \infty }^{ + \infty } \varphi \left( {\frac{u - t}{s}} \right)g\left( u \right)du,$$

where $W\left( {s,t} \right)$ is the wavelet coefficient, $g\left( u \right)$ is the time series and $\varphi$ is the Morlet mother wavelet, scaled by factor $s$ and translated in time by $t$. The Morlet mother wavelet is defined by the equation:

$$\varphi \left( u \right) = \frac{1}{{\sqrt[4]{\pi }}}\exp \left( { - i2\pi u} \right)\exp \left( { - 0.5u^{2} } \right),$$

where $i = \sqrt { - 1}$. The reason for using the Morlet wavelet is its good localization of events in time and frequency due to its Gaussian shape^18^. The wavelet coefficients are complex numbers in the time–frequency plane when the Morlet wavelet is used:

$$X\left( {\omega_{k} ,t_{n} } \right) = X_{k,n} = a_{k,n} + ib_{k,n} .$$

They define the instantaneous relative phase,

$$\theta_{k,n} = \arctan \left( {\frac{{b_{k,n} }}{{a_{k,n} }}} \right),$$

and the absolute amplitude,

$$\left| {X_{k,n} } \right| = \sqrt {a_{k,n}^{2} + b_{k,n}^{2} } ,$$

for each frequency and time.

During the measurement, phase modulations could be created, and an analysis methods to find the relationship between the phase of two signals is the wavelet phase coherence (WPCO). WPCO enables us to determine whether the oscillations detected are significantly correlated over time. To estimate the WPCO, we used the following expression^23^:

$$C_{\theta } \left( {f_{k} } \right) = \frac{1}{n}\left| {\mathop \sum \limits_{t = 1}^{n} \exp \left[ {i\left( {\theta_{2k,n} - \theta_{1k,n} } \right)} \right]} \right|,$$

where $\theta_{k,n} = \arctan \left( {\frac{{b_{k,n} }}{{a_{k,n} }}} \right)$ are instantaneous phases at each time ${\text{t}}_{{\text{n}}}$ and frequency $f_{k}$ for both signals. When two oscillations are unrelated (related), their phase difference continuously changes (remain constant) with time, thus their $C_{\theta } \left( {f_{k} } \right)$ approaches zero (one).

Additionally, we can calculate the phase difference $\Delta \theta_{k}$, which provides information about the phase lag of one oscillator compared to the other:

$$\Delta \theta_{k} = \arctan \left( {\frac{{\frac{1}{n}\mathop \sum \nolimits_{t = 1}^{n} \sin \left( {\Delta \theta_{2k,n} - \Delta \theta_{1k,n} } \right)}}{{\frac{1}{n}\mathop \sum \nolimits_{t = 1}^{n} \cos \left( {\Delta \theta_{2k,n} - \Delta \theta_{1k,n} } \right)}}} \right),$$

where $$\Delta \theta_{k} \in \left( { - 180^\circ , 180^\circ } \right).$$

### Statistical analysis

To avoid the assumption of normality in the results, the nonparametric statistical tests were used (Wilcoxon rank sum test) for all comparisons. The results of our calculations are found in Fig. 2.

To test whether the estimated values of phase coherence are statistically significant or not, the surrogate data testing method was used^17^. As we know, there are naturally less cycles of oscillations the lower the frequency^24^. This can artificially increase a wavelet phase coherence at low frequencies, even in cases where there is none^3,24^. The surrogate analysis helps to find a significance level (surrogate threshold) above which the phase coherence may be regarded as physiologically meaningful^17^. Surrogates, combined with appropriate discriminating statistics, provide a ‘statistical zero’, a threshold that is calculated from a range of data sets that definitely do not possess the property that is being investigated^3,17^. To estimate significance level in this study, an inter-subject surrogate was used^25^, which assumes that the signals collected from different subjects must be independent while having similar characteristic properties^17^. The actual value of phase coherence obtained at each frequency can then be compared with the surrogate threshold^3,17^. When the phase coherence is located above the threshold it is considered to be statistically significant^3,17,24^. In many applications of surrogates, it has been assumed that the distribution of the discriminating statistics calculated in a set of surrogates is Gaussian^17,24^, and thus the surrogate threshold has been set as a number of standard deviations above or below the mean, usually two^17,24^. However, the distribution of these surrogates is not always Gaussian thus alternatively, and more robustly, the surrogate threshold is determined as, for example, the 95th percentile of the surrogates^17^. Similar way of estimation of surrogate was used during previous studies by Gruszecki et al.^3^. Detailed description of surrogate method was recently presented by Lancaster et al.^17^.
